# Validation of a Method for the Assessment of Urinary Neopterin Levels to Monitor Health Status in Non-human-primate Species

**DOI:** 10.3389/fphys.2017.00051

**Published:** 2017-02-06

**Authors:** Verena Behringer, Jeroen M. G. Stevens, Fabian H. Leendertz, Gottfried Hohmann, Tobias Deschner

**Affiliations:** ^1^Department for Primatology, Max Planck Institute for Evolutionary AnthropologyLeipzig, Germany; ^2^Department of Biology, University of AntwerpAntwerp, Belgium; ^3^Center for Research and Conservation, Royal Zoological Society of AntwerpAntwerp, Belgium; ^4^Epidemiology of Highly Pathogenic MicroorganismsBerlin, Germany

**Keywords:** bonobo, chimpanzee, disease, sickness, biomarker, urine, validation

## Abstract

Determining individual health status is of great importance for a better understanding of life history trade-offs between growth, reproduction, and maintenance. However, existing immunological methods are invasive and therefore not suitable for investigating health status in wild populations. Thus, there is an urgent need for non-invasive methods to assess the immune status of animals. Neopterin is involved in the cell-mediated pathway of the immune response (Th1–type), secreted during the activation of monocytes and macrophages. We investigated if urinary neopterin could serve as a biomarker of health status in bonobos and chimpanzees. First, we performed a chemical validation of a commercial neopterin enzyme immune assay (EIA) for bonobo and chimpanzee urine. We then examined if urinary neopterin levels in bonobos increase during the acute period of respiratory infections. We found that neopterin levels can be reliably measured in urine of the two species with a commercial EIA. Stability experiments revealed considerable changes in urinary neopterin levels in relation to multiple freeze–thaw cycles and extended exposure to room temperature. Exposure to sunlight led to a degradation of urinary neopterin, whereas sample storage up to 2 years did not affect urinary neopterin levels. There was no detectable diurnal variation in neopterin levels, and levels remained very stable across several days in healthy individuals. While urinary neopterin levels were independent of sex, non-adult individuals had higher urinary neopterin levels than adults. Most importantly, there was a significant increase in urinary neopterin levels during a period of respiratory infection. Our results demonstrate that regular urine sample collection would allow for the monitoring of individual health status and disease progression with minimal disturbance of the subjects. In combination with behavioral, life history, and endocrinological parameters, the method can be used to investigate questions related to immunocompetence handicaps or life history trade-offs.

## Introduction

Understanding life history trade-offs between growth, reproduction, and the maintenance of health is of major importance in the field of integrative biology (Stearns, 1992). The maintenance of a well-functioning immune system is essential for resistance to infections and ultimately for survival (French et al., 2009). Therefore, determining individual health status is important for a better understanding of the course of diseases, intra- and interspecific transmission, and ultimately the effects of health status on fitness (Lazzaro and Little, 2009).

Immunology has traditionally been studied in laboratory animals with a focus on proximate mechanisms and functionality of the immune system (Pedersen and Babayan, 2011). The laboratory environment permits highly controlled conditions, facilitates repeated testing, variation of environmental parameters, and the manipulation of infections (Lazzaro and Little, 2009; Pedersen and Babayan, 2011), thereby excluding co-factors and allowing for a clear link between factors analyzed. While results of laboratory tests can be informative in terms of the trade-offs between activation of immune function on the one hand and growth and reproductive performance on the other hand. Such tests do not account for the effects of other parameters that are of importance in natural populations such as individual coevolution of pathogens and the host's immune response under conditions of limited access to resources, infection risk, and genetic diversity (Sheldon and Verhulst, 1996; Lazzaro and Little, 2009; Pedersen and Babayan, 2011).

Ecoimmunological studies on non-human primates have aimed at the ecology of, for example, infectious diseases (Chapman et al., 2005; Nunn, 2012), the spread of sexually transmitted diseases (Nunn et al., 2014), parasite and disease transmission (Côté and Poulin, 1995; Bonnell et al., 2010), as well as transmission between humans and wildlife (Daszak et al., 2000; Köndgen et al., 2008). Due to the lack of non-invasive techniques for the determination of the immune status, patterns of immune response are comparatively less well-studied in wild and captive non-human primates. So far, the health status of captive and wild primates was mainly assessed invasively by measuring immune parameters in blood samples (Eberl et al., 2001; Howell et al., 2003; Prall et al., 2015). While this approach facilitates the use of analytical techniques that have been developed for human research, the manipulation of animals for blood sampling increase the risk of injury. Furthermore, because of its invasive nature, this approach is not suitable for longitudinal studies such as the long-term monitoring of changes in parasite load, fluctuation of disease transmission, and age-related changes in immune competence, especially in wild-living animals. Thus, there is an urgent need for techniques that can non-invasively assess the immune status of animals.

Previously, non-invasive assessment of health status in non-human primates was carried out by, e.g., visual inspection (Archie et al., 2012), or by quantification of parasitic load (Gillespie et al., 2005). Urinalysis was used in non-human primates to diagnose urologic conditions or diseases of the kidneys (Simmerville et al., 2005). In urine of wild chimpanzees, urinalyses were performed using dipsticks (Kaur and Huffman, 2004; Kelly et al., 2004; Krief et al., 2005; Leendertz et al., 2010). Unfortunately, the results of dipstick analyses alone turned out to be an unreliable method for assessing the health status of these animals, because the concentration of proteins in the urine was not associated with obvious external signs of illness (Kaur and Huffman, 2004; Leendertz et al., 2010).

An approach that has received far less attention thus far in the context of studying diseases non-invasively in non-human primates is the activation of the immune response which plays a key role in fending off diseases and infections (Murr et al., 2002). The non-specific immunity or innate immunity responds to pathogens without establishing a long-lasting or protective immunity to the host. Parts of the specific and non-specific immune responses are T–cells, including the T helper cells (Th–cells). After antigen recognition, the Th–cells release interleukin 12 (IL–12), which activates the T lymphocytes type 1 and natural killer cells to release interferon–γ. In conjunction with interferon–α, other cytokines, and endotoxins, interferon–γ stimulates the activation of monocytes/macrophages. When stimulated, these cells release neopterin into body fluids. Therefore, an eventual increase in neopterin levels occurs whenever T helper cells have been activated. In humans, this cell-mediated (Th1–type) pathway of immune response can be monitored by measuring changes of neopterin levels (Huber, 1984; Fuchs et al., 1988; Murr et al., 2002). Neopterin, 2–amino–4–hydroxyl–6–(D–erythro-1′, 2′, 3′–trihydroxypropyl)—pteridine, belongs to the class of aromatic pteridines. Unlike interferon–γ, which quickly degrades and has a tendency of binding to pathogens, neopterin is chemically stable and excreted in an unchanged form by the kidneys into urine (Berdowska and Zwirska-Korczala, 2001). Because neopterin is constantly present in all body fluids, it can also be measured in urine samples (Murr et al., 2002), which can be collected non-invasively from wild and captive animals. During the course of a virus infection, neopterin levels start to decline after specific antibodies against the antigen are measurable, and stay at baseline levels when the immune system has successfully defeated the infection (Murr et al., 2002). Therefore, changes in neopterin levels are closely associated with the activation of the early cellular immune response (Hamerlinck, 1999; Pingle et al., 2008).

In humans, an increase in serum and urinary neopterin levels can be observed in patients with various inflammatory diseases during the acute phase of infection, e.g., acute viral (such as hepatitis and rubella) and intracellular bacterial infections (such as pulmonary tuberculosis and leprosy) as well as in patients with chronic infections or tumors (Huber, 1984; Hamerlinck, 1999; Widner et al., 1999; Schennach et al., 2000). In summary, neopterin measurement provides an insight into cell-mediated immune response and allows for the monitoring of disease progression (Berdowska and Zwirska-Korczala, 2001). As neopterin is involved in the non-specific immune response, it can be used as a marker to identify the health status of individuals for which the exact disease is not yet identified. In this study, we evaluate the potential practicality of non-invasive measures of neopterin for monitoring immune system functioning in urine samples of bonobos (*Pan paniscus*) and chimpanzees (*Pan troglodytes*). We tested whether neopterin can be reliably measured in urine samples of the two ape species. Furthermore, we investigated the stability of urinary neopterin at room temperature, when urine samples were stored at minus 20°C for 2 years (long-term storage), after repeated freeze-thaw cycles, and after exposure to sunlight. In addition, we explored if a diurnal pattern in urinary neopterin excretion exists as well as how stable urinary neopterin levels are in healthy individuals during a week, and we investigated sex and age effects on urinary neopterin levels. Finally, we analyzed the diagnostic potential of urinary neopterin levels comparing samples from the same bonobo during a period of respiratory disease with samples collected during a period of health.

## Materials and methods

### Ethics statement

All urine samples were collected non-invasively. The study was carried out in accordance with NIH published standards. The protocol for urine sample collection was approved by the authorities of each zoo. All animals were housed in social groups in European zoos. The apes received a mix of fruits and vegetables several times per day and had *ad libitum* access to fresh water.

### Animals and sample collection

We used 66 samples from 21 male and female bonobos and 10 samples from male and female chimpanzees, collected in 11 different zoos (Table 1). The chimpanzees ranged from 18 to 49 years of age and the bonobos ranged from 2 to 52 years (Table 1). Urine samples were randomly collected throughout the day. Samples were collected by the keepers directly from the urine stream or taken off the ground, when the individual could be identified and when contamination with feces could be excluded. For detailed description see Behringer et al. (2014).

**Table 1:** **Species, zoo, number of urine samples, and purpose of use for measuring urinary neopterin**.

**Species**	**Sex**	Age^*^	**Zoo**	**No. of samples**	Used^**^
Bonobo	f	9, 10	Planckendael	5	h, da, w
Bonobo	f	18, 19	Planckendael	6	h, da, w
Bonobo	f	35	Stuttgart	1	s
Bonobo	f	25	Frankfurt	1	d
Bonobo	f	27, 28	Planckendael	6	h, da, w
Bonobo	f	5, 6	Planckendael	6	h, da, w
Bonobo	f	52	Frankfurt	1	s
Bonobo	f	12	Frankfurt	1	se
Bonobo	f	2	Planckendael	4	h, da, w
Bonobo	f	5	Frankfurt	1	se
Bonobo	f	30	Leipzig	1	s
Bonobo	f	14, 15	Frankfurt	3	h, d, r
Bonobo	m	7, 8	Planckendael	6	h, da, w
Bonobo	m	11, 12	Frankfurt	5	d, h, s, se, r
Bonobo	m	21	Leipzig	1	s
Bonobo	m	28	Frankfurt	2	d, s
Bonobo	m	14, 15	Planckendael	6	h, da, w
Bonobo	m	7	Frankfurt	2	h
Bonobo	m	4	Frankfurt	2	h, r
Bonobo	m	2	Frankfurt	1	se
Bonobo	m	18, 19	Planckendael	5	h, da, w
Chimpanzee	f	38	Halle	1	s, r
Chimpanzee	f	46	Aalborg	1	d
Chimpanzee	f	38	Heidelberg	1	s
Chimpanzee	f	11	Badoca	1	d
Chimpanzee	f	39	Heidelberg	1	s
Chimpanzee	m	18	Augsburg	1	s, r
Chimpanzee	m	19	Badoca	1	d
Chimpanzee	m	25	Copenhagen	1	d
Chimpanzee	m	49	Kittenberger	1	s, r
Chimpanzee	m	37	Halle	1	s

* *Age, age at sample collection*.

** *d, dilution; da, daily variation; h, health status; s,stability test; se, sunlight exposure; r, re-measurement after 2 years; w, weekly comparison*.

Urine samples were collected and experiments were carried out between February 2012 and August 2016. Samples of both species were used to test the effects of different ambient conditions on urinary neopterin levels including: the freeze-thaw cycles, storage at room temperature, long-term storage for 2 years, and exposure to sunlight. Samples used for these experiments were collected throughout the day (between 7:00 and 18:00) and came from subjects living in different zoos (Table 1). In detail, the freeze-thaw experiment was performed to assess urinary neopterin level stability in the samples. We pooled urine samples of three males and three females from each species, took five aliquots from each pooled sample, and exposed them to an increasing number (1–5) of freeze-thaw cycles. To examine the degradation of urinary neopterin over a period of 48 h at room temperature (23°C), two pooled samples were prepared as described above. Five aliquots were kept at room temperature, and then frozen after 1, 4, 8, 24, and 48 h, respectively. To assess degradation after long-term storage (at −20°C), aliquots of the seven original samples measured in July 2014 were measured again in July 2016. For the sunlight experiment, aliquots of 200 μl urine from four individuals were kept for 3 h (a) in the dark at room temperature, (b) at artificial light at room temperature, (c) at direct sunlight, and (d) at sunlight on ice packs to exclude temperature effects.

Urine samples from bonobos were used to assess diurnal variation, weekly changes, sex- and age-specific variation, as well as within individual comparison of urinary neopterin levels when being sick vs. healthy (Table 1). To assess diurnal variation, samples from eight bonobos were collected in Planckendael Zoo at 7:00, 12:00, and 17:00, and for temporal changes twice in 1 week at 7:00. To evaluate the impact of age and sex, samples of healthy bonobos (N_females_ = 7, N_males_ = 6) were collected in Planckendael and Frankfurt Zoo. For investigating the impact of disease on urinary neopterin levels, we conducted a within subject comparison of 22 bonobos housed in Planckendael and Frankfurt Zoo during periods when the individuals were either healthy or sick (Table 1). Urine was collected opportunistically during a period of illness (sick sample) when the animals were diagnosed with an unspecific respiratory disease accompanied by running noses and coughing. During the period when symptoms were visible, one sample was collected from each individual at Frankfurt Zoo between the 11^th^ and 12^th^ March 2014, and at Planckendael Zoo between the 27^th^ of February and the 1^st^ of March 2013. Although daily documentation by zookeepers verified that all animals showed symptoms of a respiratory disease, it was not possible to reproduce the exact time course of the disease for each animal. After convalescence, we collected an additional sample from the same individual (healthy sample).

After collection, all urine samples were stored at −20°C in the zoos and transported frozen to the Max Planck Institute for Evolutionary Anthropology (MPI-EVA), Leipzig, Germany. At the MPI-EVA samples, were stored at −20°C until analysis. More detailed information on urine sample collection can be found in Behringer et al. (2014).

### Urinary neopterin measurement with an ELISA

To measure neopterin levels in urine of bonobos and chimpanzees, we used a commercial competitive neopterin ELISA (Neopterin ELISA, Ref. RE59321, IBL International GmbH, Hamburg, Germany), which was created for the quantification of neopterin in human serum, plasma, and urine. For analyses, all urine samples were thawed, vortexted, centrifuged, and diluted 1:100 with the assay buffer of the supplier. The assay was performed following the instructions from the supplier. We took 20 μl of the diluted urine, 100 μl of the enzyme conjugate, and added 50 μl of the neopterin antiserum to each well on the plate. The plate was incubated in the dark for 90 min. After incubation, plate was washed four times with washing buffer, and 150 μl of tetramethylbenzidine substrate solution was added. The reaction was stopped after 10 min. of incubation with 150 μl of the provided stop solution. Optical density was measured photometrically at 450 nm. All samples, standards, and controls were measured in duplicate. Inter-assay variation of four separate runs for high- and low-quality controls was 6.2 and 7.7%, respectively. Intra-assay variation was 6.1% (*N* = 42 samples). Results are expressed in nmol/L.

To compensate for variation in volume and concentration of the collected urine, specific gravity (SG) was measured with a digital handheld refractometer (TEC, Ober-Ramstadt, Germany). SG population average for the measured bonobo urine samples was 1.0095. No correction factor was needed for the chimpanzee urine, because the results were only used for within sample comparison. The correction for neopterin concentration for each bonobo sample was calculated as described in Miller et al. (2004). Final results are expressed in urinary neopterin (nmol/L) corrected for SG (corr. SG).

### Assay validation

To test the reliability of neopterin measurements in urine samples, the assay was validated by (a) parallelism and (b) an accuracy test for each species.

- For assessment of parallelism, four samples from each species (two males and two females) were pooled and serially diluted with the provided buffer of the assay kit (25-, 50-, 100-, 200-, and 400-fold).
- To assess the assay accuracy of the neopterin measurement, we pooled six samples (three females and three males) from each species. Each pool sample was spiked with 1.35, 4, and 12 nmol/l of standard provided by the assay kit, respectively.

### Statistical analysis

To assess the effect of sampling day (samples collected twice per week), long-term storage (freezing for 2 years), and for comparing urinary neopterin levels in samples collected during healthy and sick periods, we ran an exact Wilcoxon test for each data set. Additionally, for the long-term storage, we ran a Spearman rank correlation on the urinary neopterin measures. To compare urinary neopterin levels between females and males we used a two tailed *t*-test. All tests were run in R (R Core Team, 2016).

## Results

### Assay validation

**Figure 1:**
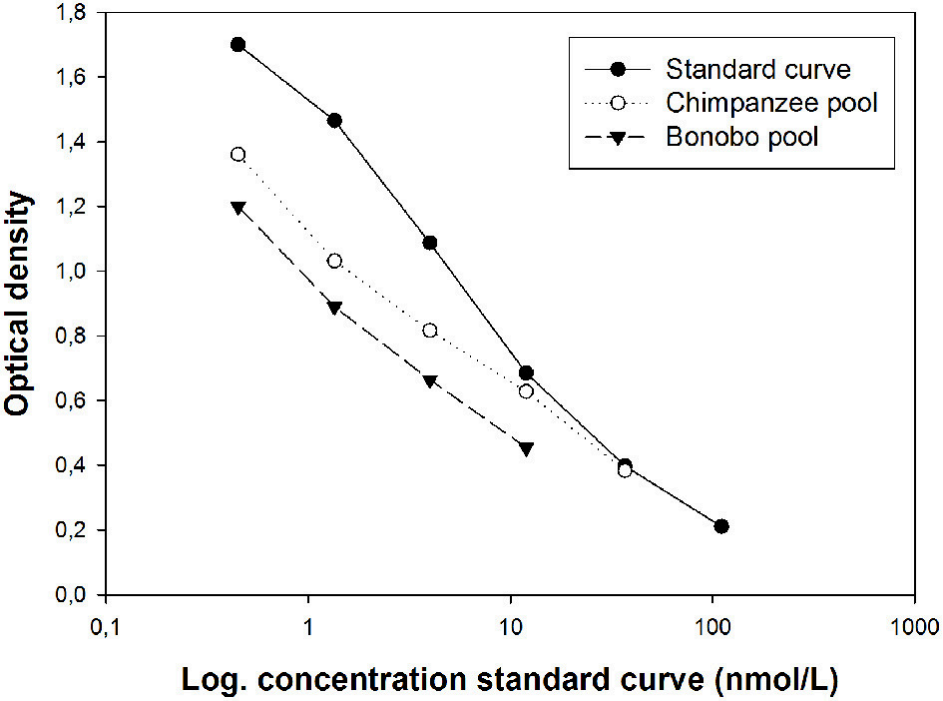
**Optical density of serial dilutions of two spiked pooled urine samples from chimpanzees and bonobos in relation to the standard curve**.

Serially diluted pooled urine samples were parallel to the neopterin standard curve in both species (Figure 1).

Average recovery of spiked urine samples was 99.3% (range: 75–114%) for bonobos and 100.3% (range: 80–122%) for chimpanzees. In both species, higher recovery was achieved in samples that were spiked with higher concentrations of neopterin (Table 2).

**Table 2:** **Accuracy of urinary neopterin measurements in pooled samples (1:100 diluted with the assay buffer) spiked with three different concentrations of neopterin**.

**Species**	**Standard**	**Neopterin [nmol/L]**		**Recovery (%)**
**Pool sample**		**Expected**	**Measured**	
Bonobo	1.35	1.74	1.99	114
	4	3.49	3.81	109
	12	8.37	6.28	75
Chimpanzee	1.35	2.25	2.74	122
	4	3.45	3.42	99
	12	8.81	7.05	80

### Stability of urinary neopterin levels in bonobos and chimpanzees

#### Freeze-thaw cycle experiment

**Figure 2:**
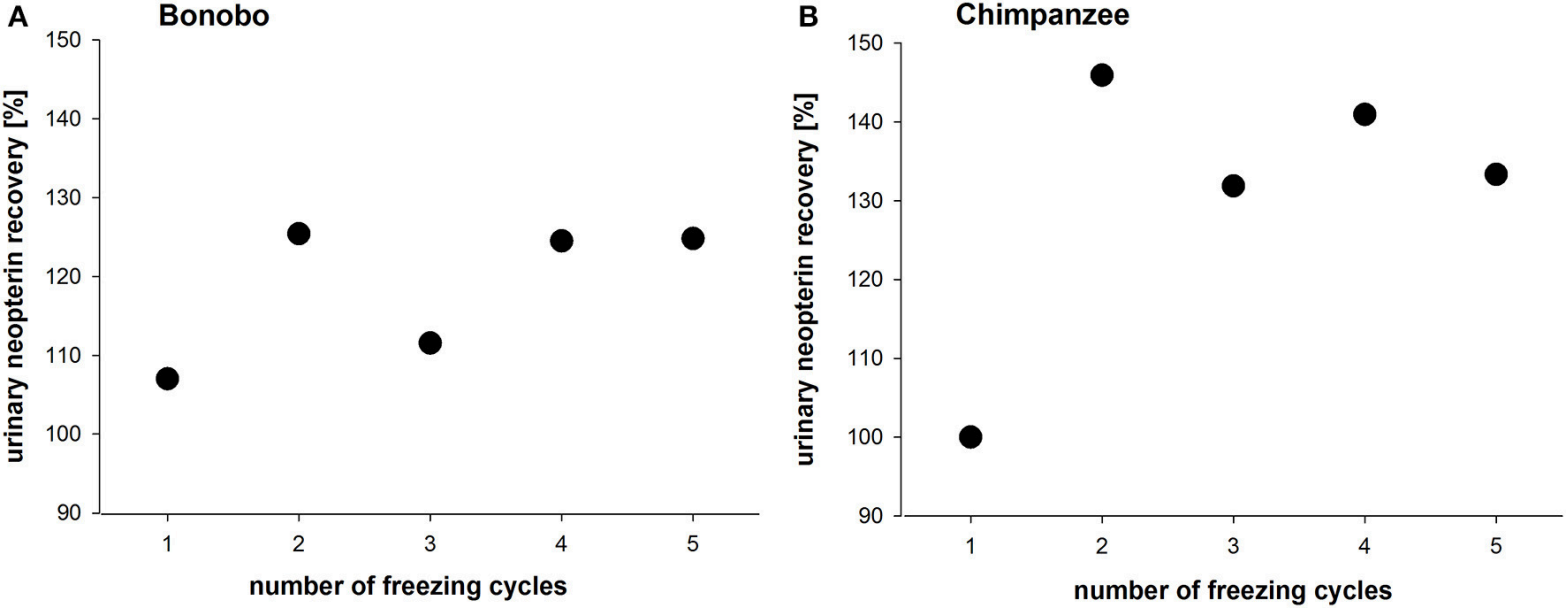
**Urinary neopterin levels in pooled samples of (A)** bonobos and **(B)** chimpanzees over the course of five freeze-thaw cycles.

Repeated freeze-thaw cycles (range: 1–5 times) of urine samples led to an increase of the original measured urinary neopterin levels in both species (Figures 2A,B). In bonobos, urinary neopterin levels increased to 120% after the first thawing cycle, then decreased to 105% after the second cycle, and returned to about 120% thereafter for the remaining cycles (Figure 2A). In chimpanzee samples, urinary neopterin levels reached 145% after the first thawing cycle and stayed between 130 and 140% for the following four cycles (Figure 2B).

#### Room temperature experiment

**Figure 3:**
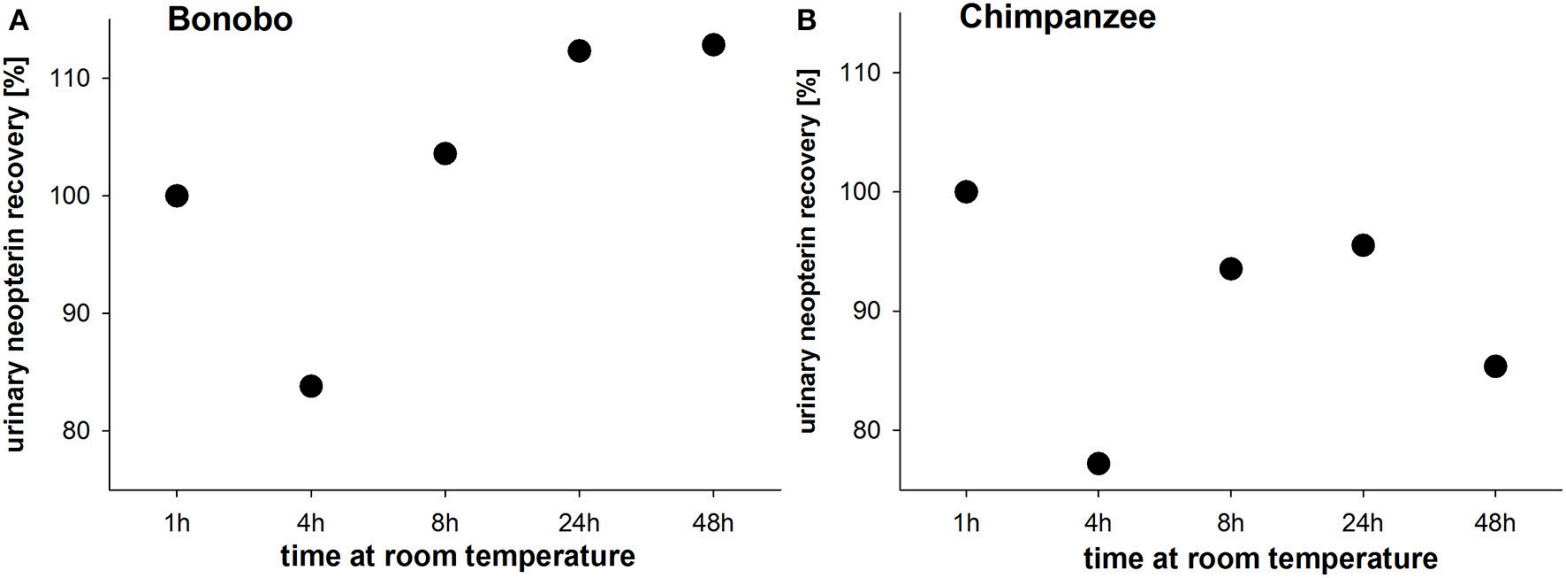
**Urinary neopterin levels in pooled samples of (A)** bonobos and **(B)** chimpanzees kept at room temperature for 48 h.

In pooled urine samples stored for 4 h at room temperature, urinary neopterin levels decreased by approximately 20% in both species (Figures 3A,B). In the bonobo pooled sample, urinary neopterin levels increased consecutively after 8 and 24 h and remained at the 24 h level thereafter (Figure 3A). In chimpanzees, urinary neopterin levels also increased after 8 h, and decreased again after 24 h (Figure 3B).

#### Long-term storage experiment

After 2 years of storage, on average 90% of the first neopterin measurement was re-measured. In one individual, urinary neopterin was degraded by more than 40% (Table 3). Overall, urinary neopterin levels at the beginning and after 2 years of storage showed no significant differences (exact Wilcoxon test: *T*^+^ = 1.69, *N* = 7, *P* = 0.144), and were significantly positively correlated (r_spearman_ = 0.96, *P* = 0.002, *N* = 7).

**Table 3:** **Neopterin measures (nmol/L) in urine samples before (first measurement) and after 2 years (second measurement) of storage at −20°C**.

**Sample no**.	**First measurement**	**Second measurement**
	**Neopterin (nmol/L)**	
1	418	292
2	644	700
3	713	714
4	3491	3153
5	1184	996
6	783	833
7	980	749

#### Sunlight exposure experiment

Urinary neopterin levels in samples stored in the dark compared with storage in artificial light changed only slightly in two of three samples (Table 4). In samples exposed for 3 h to sunlight, the neopterin concentration was not measurable any more, even when the samples were cooled during this period (Table 4).

**Table 4:** **Neopterin levels (nmol/L) in four urine samples after storage for 3 h in the dark, artificial light (light), sunlight with cool packs (sun and cool), and sunlight without being cooled (sun)**.

**Sample**	**1**	**2**	**3**	**4**
**Condition**	**Neopterin (nmol/L)**			
Dark	129	3618	1294	2450
Light	197	3666	1218	2547
Sun and cool	Too low	Too low	Too low	Too low
Sun	Too low	Too low	Too low	Too low

#### Diurnal variation

**Figure 4:**
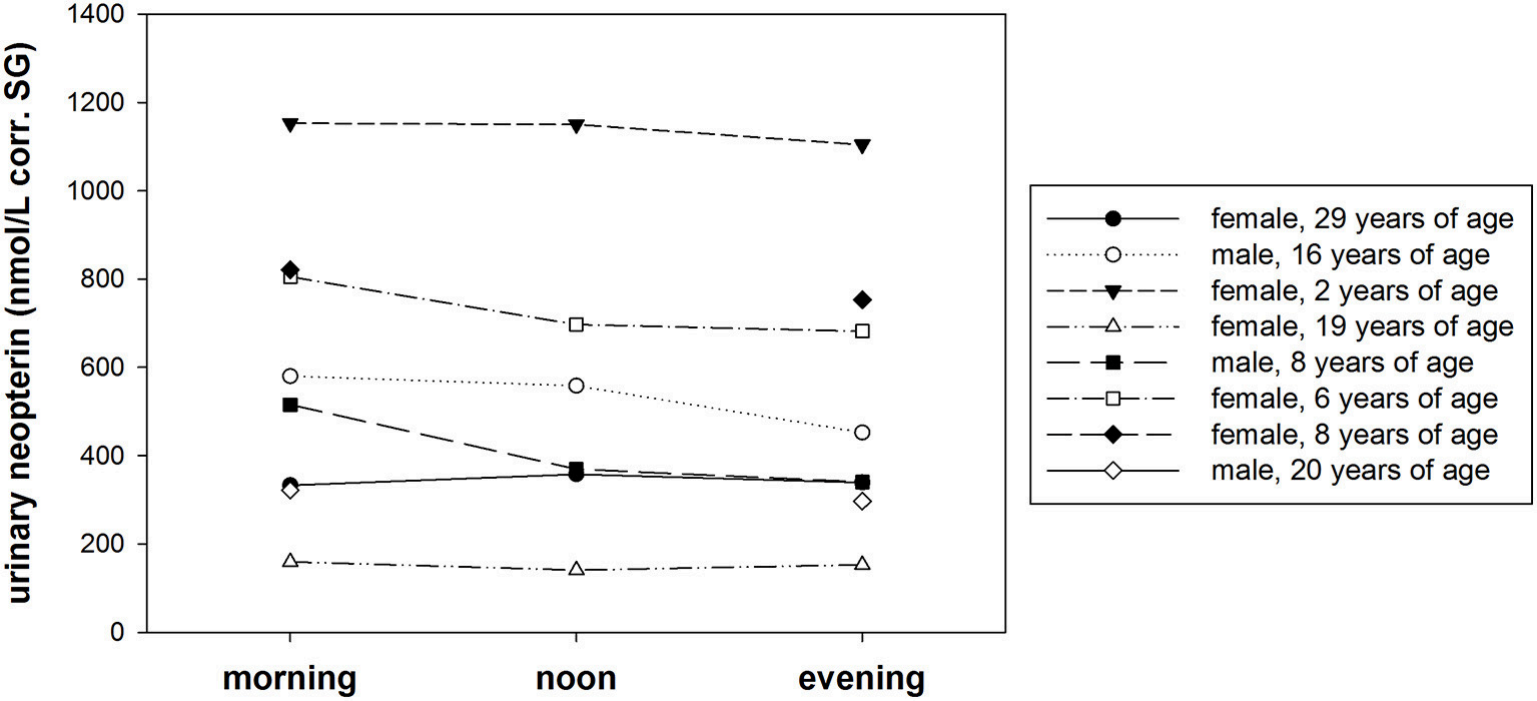
**Variation of urinary neopterin levels (nmol/L) corrected for specific gravity (corr. SG) collected at 7:00 h (morning, ***N*** = 8), at 12:00 h (noon, ***N*** = 5), and at 17:00 h (evening, ***N*** = 8) from eight different bonobos**.

Urinary neopterin levels in samples of eight bonobos showed no obvious diurnal pattern (Figure 4). The largest effect size was in one adult male whose urinary neopterin level was approximately 20% lower in the evening (452.2 nmol/L corr. SG) compared with the morning sample (579.8 nmol/L corr. SG). The average effect size was 1.1 between morning/noon, morning/evening, and noon/evening. Urinary neopterin levels differed between individuals, and ranged from 323 nmol/L corr. SG to 1153.1 nmol/L corr. SG.

#### Weekly changes

**Figure 5:**
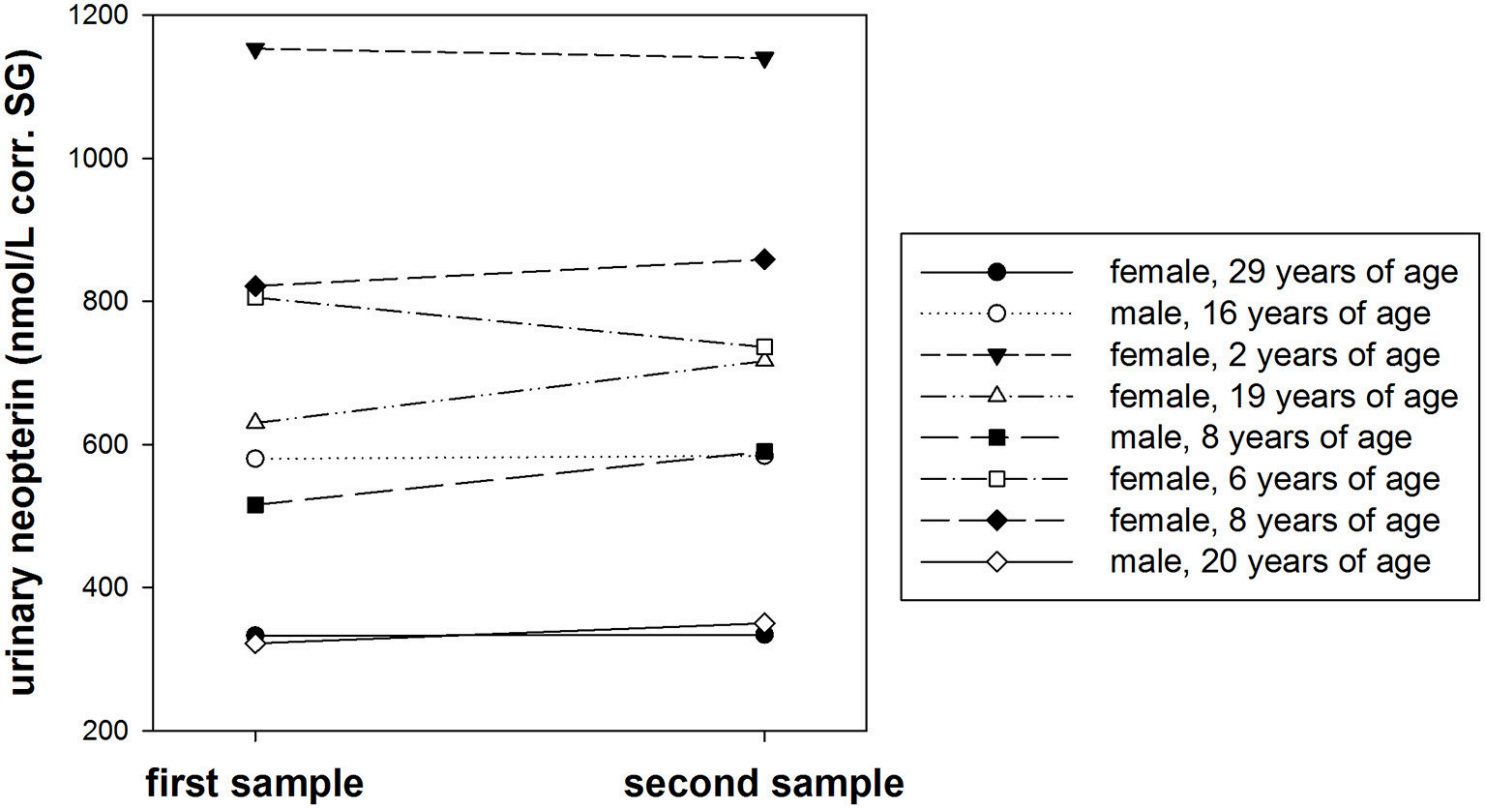
**Urinary neopterin levels (nmol/L) corrected for specific gravity (corr. SG) in samples from eight bonobos collected twice in 1 week**.

Urinary neopterin levels showed no significant differences over the course of 1 week, consistent across eight different individuals (*T*^+^ = −1.071, *df* = 7, *P* = 0.320, Figure 5). The average urinary neopterin level change was 4.1% (range: 0.4–14.5%).

#### Sex and age comparison

There was no significant difference in urinary neopterin levels of healthy male (*N* = 6) and female (*N* = 8) bonobos (*T* = 0.7532, *df* = 10.445, *P* = 0.468), but the three youngest individuals (2, 6, and 8 years of age) had the highest levels.

#### Comparison of urinary neopterin levels in sick and healthy bonobos

**Figure 6:**
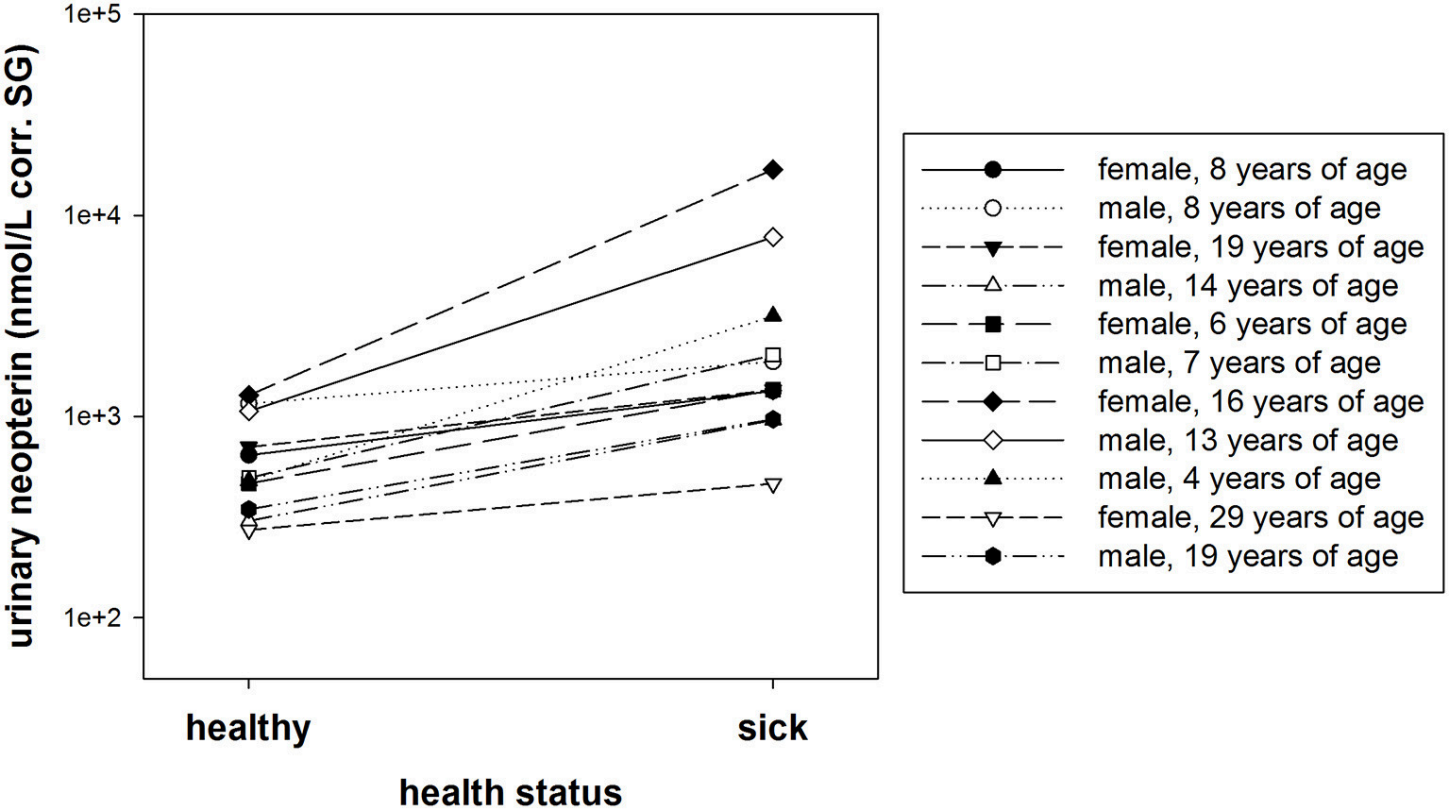
**Urinary neopterin levels (nmol/L) corrected for specific gravity (corr. SG) from 11 individuals collected at a time of acute illness and a time without symptoms**. The y-axis is displayed on a log scale.

A within individual comparison of urinary neopterin levels (corr. SG) during healthy and sick periods showed significantly higher urinary neopterin levels in bonobos with symptoms of a respiratory disease (exact Wilcoxon test: *T*^+^ = 66, *N* = 11, *P* < 0.001, Figure 6).

We found that both average and median values of urinary neopterin levels (nmol/L corr. SG) were higher in sick bonobos than in healthy bonobos (Table 5). However, the variation in urinary neopterin levels was greater in sick compared to healthy individuals (presented by SD., Min., and Max. in Table 5). The average effect size was 4.8 between healthy and sick neopterin levels. The strongest effect size between the two conditions was found in an adult female (effect size of urinary neopterin levels: sick 13.3 times higher vs. healthy), and the smallest in an 8-year-old male (effect size of urinary neopterin levels: sick 1.6 times higher vs. healthy).

**Table 5:** **Description of urinary neopterin levels (nmol/L corr. SG) in 11 bonobos during healthy and sick periods, as well as the effect sizes between the two conditions**.

	**Neopterin (nmol/L corr. SG)**		**Effect size**
	**Healthy**	**Sick**	
Mean	655.0	3474.4	4.8
SD.	355.4	4891.9	
Median	494.9	1356.6	3.2
Max.	1274.8	16913.9	13.3
Min.	273.1	463.6	1.6

## Discussion

This study demonstrates that neopterin levels can be reliably measured in urine of bonobos and chimpanzees. The stability experiments revealed that urinary neopterin levels increased during multiple freeze-thaw cycles in both species. Additionally, storing samples at room temperature for 4 h decreased urinary neopterin levels in both species, but levels were unaffected by 2 years of long-term storage in the freezer. After 3 h of exposure to sunlight, urinary neopterin levels were degraded below the sensitivity threshold of the assay. The time of urine collection during a day had no effect on neopterin levels; moreover, within a 1-week period there was only minimal variance in the urinary neopterin levels within an individual. While urinary neopterin levels were independent of sex, age had an effect, with higher urinary neopterin levels in samples from young individuals. However, this effect should be statistically tested with a larger sample size in future studies. Finally, while urinary neopterin levels of healthy and sick individuals overlapped, there was a significant increase of urinary neopterin levels during a time of a respiratory infection.

We validated a commercial assay originally developed to measure neopterin in human serum and urine. We extended the validation of the assay kit for bonobos and chimpanzees. This validation for each species is an essential requirement for measuring physiological markers extracted from organic substances like urine or fecal samples (Buchanan and Goldsmith, 2004). The chemical validation, including serially diluted urinary neopterin and recovery of spiked urine samples, revealed that neopterin can be reliably measured in urine samples of bonobos and chimpanzees.

The second part of the study explored the stability of neopterin in urine samples of bonobos and chimpanzees with changing ambient conditions. It is essential to explore the effect of different ambient conditions, because biological markers in samples are vulnerable to degradation (Buchanan and Goldsmith, 2004). We found that in samples from both species, urinary neopterin levels increased with the number of freeze-thaw cycles. In human serum, neopterin levels are also known to be affected by frequent thawing cycles, and in macaques, urinary neopterin significantly increased after three freeze-thaw cycles (Heistermann and Higham, 2015). Therefore, we recommend to avoid freeze-thaw cycles of the samples or at least to keep the number of freeze-thaw cycles constant for all samples.

When urine samples were kept at room temperature for a period of 24 or 48 h, urinary neopterin levels first decreased and then increased in samples from both species. In a study on macaques, urinary neopterin levels in samples exposed to room temperature for 21 days increased gradually and elevation reached a significant level at the second day of exposure to room temperature (Heistermann and Higham, 2015). Therefore, we recommend freezing the samples promptly after sample collection and the preparation of dilutions. In terms of long-term storage, the neopterin assay kit manual (Neopterin ELISA RE59321) informs that urine samples could be stored for 6 month at −20°C. After macaques urine was stored for an 8-month period, urinary neopterin levels showed no statistical degradation (Heistermann and Higham, 2015). We extended the storage period to 2 years at −20°C, and found that urinary neopterin levels remained stable even after 2 years of storage at −20°C. These results indicate that urinary samples for neopterin measurement could be stored at −20°C for extended periods of time without risking degradation. One parameter that did lead to degradation of neopterin levels was sunlight (Müller et al., 1991; Laich et al., 2002). In our experiment, 3 h of exposure to sunlight were sufficient to degrade neopterin below the assay's sensitivity threshold. Accordingly, for studies aiming at measuring urinary neopterin levels, we recommend avoiding exposure of urine samples to sunlight, many repeated freeze-thaw cycles, and sample storage at room temperature.

We found no clear pattern of diurnal variation in urinary neopterin levels corroborating findings of other studies that indicate that neopterin is produced and released in a remarkably constant proportion (Murr et al., 2002), and that therefore, daytime of sampling is trivial. Furthermore, we found only modest variation of urinary neopterin levels across a 4-day period. This confirms findings from a study on adult humans that showed that serum neopterin levels were stable throughout 1 year, but increased during an influenza infection and afterwards returned to baseline levels (Wachter et al., 1989). Consistent with other studies in humans (Werner et al., 1987; Müller et al., 1991; Diamondstone et al., 1994), and macaques (Higham et al., 2015), we found that neopterin levels did not vary with sex, but that there was considerable variation across healthy adult individuals. Overall, this indicates that healthy individuals have stable baseline neopterin levels; however, the causes for inter-individual variation of urinary neopterin baseline levels remain to be explored.

Our finding that younger individuals (less than 9 years of age) had the highest urinary neopterin levels is in line with data from human studies showing that urinary neopterin levels were higher in subjects younger than 18 years of age (Werner et al., 1987), and that the highest neopterin levels were measured in neonates (Müller et al., 1991). Young children are facing an elevated infection risk when attending daycare center, kindergarten and school (Wald et al., 1991; Mikolajczyk et al., 2008). These challenges to the immune system, when children come more into close physical contact with other children, lead to frequent increases in urinary neopterin levels (Winkler et al., 2003). In young chimpanzees, social play and the number of play partners increase in the first 3 years of life. Therefore, the risk of infection increases in this life stage (Kuehl et al., 2008), which could potentially lead to the higher neopterin levels described in this study. However, the density of sample collection and numbers of individuals does not allow us to make any firm conclusions.

In our study, there was no sex difference in urinary neopterin levels corrected for specific gravity. This result is in line with serum neopterin studies in healthy humans (Reibnegger et al., 1988; Müller et al., 1991; Diamondstone et al., 1994; Satoh et al., 1998) and macaques (Higham et al., 2015). Results of studies investigating human urinary neopterin levels are ambiguous. While some found no sex difference (Werner et al., 1987; Hamerlinck, 1999), others found higher urinary neopterin levels in women than in men (Hausen et al., 1982; Mura et al., 1984). However, this sex difference is most probably an effect of the correction of the urinary neopterin levels with creatinine. Urinary creatinine levels correlate with muscle mass, therefore, since men have higher muscle mass, they also have higher urinary creatinine levels which lowers their corrected neopterin levels (Hausen et al., 1982; Fuchs et al., 1984; Reibnegger et al., 1986). As with other biomarkers, when investigating sex differences in urinary levels, a concentration correction with specific gravity should be preferred over creatinine (Miller et al., 2004; Emery Thompson et al., 2012).

During periods of unspecific respiratory infections, neopterin levels in bonobo urine were significantly elevated in our study. These results are consistent with prior studies, in which changes in neopterin levels were used to track viral infection in macaques. In macaques, urinary neopterin level increased after artificial viral infection (Fendrich et al., 1989; Higham et al., 2015). In human patients with pulmonary tuberculosis, researchers found a correlation between mean neopterin levels and the extent of disease (Fuchs et al., 1984). Furthermore, every disease associated with intensified monocyte/macrophage response is accompanied by increased neopterin levels (Berdowska and Zwirska-Korczala, 2001). Previous studies have shown that neopterin levels undergo dramatic changes during the course of an infection (Murr et al., 2002) and therefore, the diagnostic value of urinary neopterin levels, e.g., the responsiveness of urinary neopterine excretion in relation to the severity of the infection and/or the strength of the immune response, depends on the timing of sampling within the period of infection and repetition of sampling throughout the entire infection period. Yet, the overlap of urinary neopterin levels from healthy and sick individuals suggests that detection of an acute infection depends on baseline samples from the same individual. Consequently, it is not possible to define a range of “healthy” neopterin levels and to distinguish them from levels indicative of sickness. Instead, detection of an infection or disease requires sampling over longer periods of time or at least solid baseline values. Therefore, regular urine sample collection would allow for the monitoring of individual health status and disease progression via neopterin level changes. In addition, the monitoring of immune status or frequency and degree of infections in individuals in their natural habitat, in combination with behavioral, life history, and endocrinological parameters can be used to investigate questions in relation to immunocompetence handicaps (Hamilton and Zuk, 1982; Folstad and Karter, 1992; Roberts et al., 2004) or life history trade-offs (French et al., 2009). Because such studies depend on repeated measures of the immune status, the non-invasive measurement of urinary neopterin levels would be the ideal method of choice.

## Conclusion

Our study demonstrates that urinary neopterin measurements can be used to monitor aspects of the health status of captive and wild-living apes with minimal disturbance of the subjects. Following a similar validation protocol, urinary neopterin measurements could become a valuable tool for non-invasive health monitoring of other wild-living animal species. However, since neopterin levels are highly variable across individuals, baseline levels from urine samples during healthy periods are needed for comparison to assess the health status of an individual. Furthermore, neopterin levels are higher in non-adult in comparison to adult individuals. To prevent degradation of neopterin, urine samples need to be frozen as soon as possible after collection, and repeated freeze-thaw cycles and sunlight exposure should be avoided.

## Author contributions

VB, FL, and TD conceived the ideas and designed methodology; VB, JS, and GH collected the data; VB and TD analyzed the data and led the writing of the manuscript. All authors contributed critically to the drafts and gave final approval for publication.

### Conflict of interest statement

The authors declare that the research was conducted in the absence of any commercial or financial relationships that could be construed as a potential conflict of interest.
